# Approaching an undergraduate medical curriculum map: challenges and expectations

**DOI:** 10.1186/s12909-021-02778-6

**Published:** 2021-06-10

**Authors:** Rima Chakrabarti, Katie Wardle, Tor Wright, Taylor Bennie, Faye Gishen

**Affiliations:** University College London, London, UK

**Keywords:** Curriculum, Curriculum map, UCLMS

## Abstract

**Background:**

Feedback received from medical students at University College London Medical School (UCLMS) suggested a lack of clarity regarding the contents and subsequent assessment of the undergraduate curriculum. In order to address these issues, a specialist team was established with the aim of designing and implementing a Curriculum Map (CM), which have been recognised in their ability to provide a centralised, visual representation of the curriculum. While multiple perspectives from educators to stakeholders can be considered here, the need for the CM to remain student centred was identified as key at UCLMS. The aim of this study was therefore to understand the requirements of the CM prior to production from the perspective of the medical students.

**Methods:**

A mixed-methods sequential study was conducted. The first stage involved gathering quantitative data using a primary online survey. This used 15 questions, rated by Likert scales and focussed around three domains: depiction of content, functionality and students’ likely engagement with a CM. There was a free-text question for additional comments. The second stage consisted of multiple student focus groups representing different years of the programme, conducted by trained facilitators following a predetermined scheme. Reflective Thematic Analysis (RTA) was used to synthesise the qualitative data, which was read independently by two researchers. All students at UCLMS were invited to participate in the study.

**Results:**

There were 409 survey responses. 92% of students said they were ‘likely’ or ‘very likely’ to use a CM, with their key intended use being to monitor their learning progress and ensure preparedness for assessments. Five key themes emerged from the focus groups, namely that students wanted a CM to be: comprehensive; simple and intuitive; able to link content throughout the course; aligned with assessment; and useful to monitor students’ progress.

**Conclusions:**

Through this study, valuable insight was gained on students’ ideal preferences for the CM. Understanding this was important in order to ensure that its co-design remained student-centred prior to its design and launch. This study also highlighted the need to set realistic expectations for students on the role of a CM in preparing them for assessments, and ultimately professional practice.

## Background

The contributions of key education scholars, including Abraham Flexner and William Osler in the early twentieth century, remain some of the most significant in shaping UK and international undergraduate medical training programmes [1, 2]. However, as the healthcare and education landscapes, focus and modes of delivery evolved dynamically, this presents on-going challenges in preparing the future workforce for the realities of working life [3]. Undoubtedly, medical schools play a crucial role in facilitating this preparation, relying on the delivery of robust and rigorous curricula that align with the outcomes outlined by the UK regulatory body, the General Medical Council (GMC) [4, 5]. However, delivering these vast and expanding curricula within the constraints of an undergraduate programme require an understanding of the different components of the curriculum as well as how these can be accessed by the primary users: medical students.

### Defining a curriculum

The theoretical definition of *‘curriculum’* is fundamental to the design of a curriculum map. The word curriculum is derived from the Latin word *‘currere’* meaning ‘to run’, literally translating as ‘running a course’ [6]. This definition has been subject to several interpretations within education. For some scholars, it is considered the “*written plan depicting the scope and arrangement of the projected educational program*” [7]. For others, its interpretation is more inclusive, involving *“the reconstruction of knowledge and experience that enables the learner to grow in exercising intelligent control of subsequent knowledge and experience*” [8].

Further sub-classifications define different components of a curriculum, such as;

- **Formal or written curriculum** – the policy documents outlining what students are expected to know
- **Taught curriculum** - the content that is delivered through educational activities for the students to learn from
- **Assessed curriculum** –the content that is examined and used to determine if student learning has fulfilled regulatory expectations [9, 10]

English [11, 12] offers an alternative conceptualisation; broadly defining curriculum as syllabus. Originally derived from the Latin word for ‘list’, a syllabus outlines the course of study, providing students with what they are expected to know and their responsibilities [13]. From an institutional perspective, a syllabus can also act as a contract, enabling both students and educators to be aware of course policies, assessment and evaluation [14]. There are limitations with a syllabus-centred definition of curriculum; this narrower conceptualisation does not necessarily account for the fact that what is being taught may not be the same as what is being learned [15]. Prideux [16] also criticises the notion of restricting the curriculum to a syllabus, as it does not acknowledge the powerful role of the *hidden curriculum* in learning. The hidden curriculum refers to the socialisation process that occurs through continued and prolonged exposure to the environment, and has gained increasing recognition in medical education for contributing to learners thinking and behaving like doctors [17, 18].

For medical schools, given the complexities in the theoretical conceptualisation and definition of the curriculum, a number of challenges are presented when considering a curriculum map’s design and delivery.

### The medical curriculum

The GMC provides a framework for the medical curriculum, defining it as “*a statement of the intended outcomes, encompassing content, teaching, learning and assessment methods, feedback and supervision as part of the educational programme*” [19]. At present, a universally agreed consensus on the design and delivery of the undergraduate medical curriculum does not exist in the UK [20, 21]. However, the GMC’s guidance ‘Outcomes for Graduates’ [5] (2018) reflects dynamic shifts in population health needs and changing societal perspectives.

The multi-disciplinary approach to disease management, coupled with an appreciation of patient enablement and choice [20, 22] have affected how healthcare is delivered in the UK and elsewhere. Topics that may not have previously been considered important within a medical curriculum, such as diversity and inclusivity, have gained prominence within medical education [5, 23]. Like other medical schools, these changes have been reflected in the evolving curriculum at University College London Medical School (UCLMS), which also includes teaching on social justice, and well-being. Recognising the importance of addressing and facilitating discussion on these issues has been particularly important during the Covid-19 pandemic. Importantly, the dynamic nature of the medical curriculum which encompasses real-world issues enables preparation of future worldly practitioners.

**Fig. 1:**
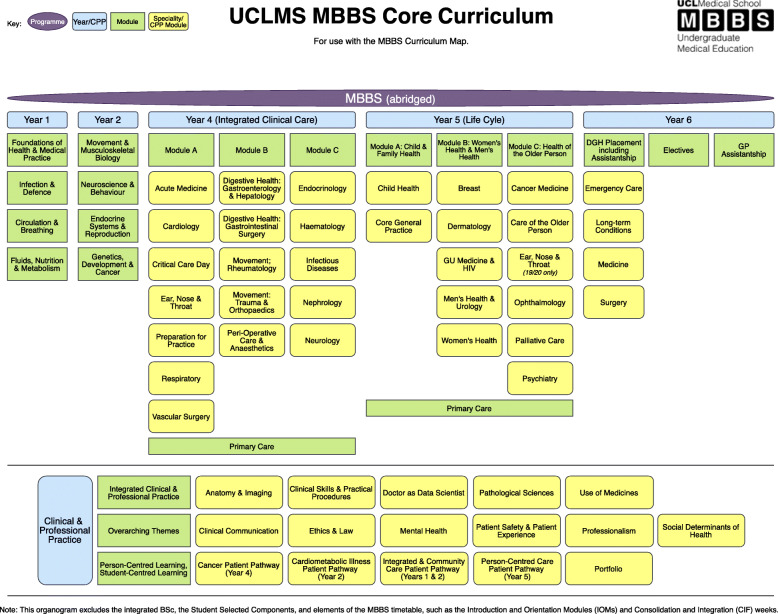
UCLMS Core Curriculum

However, the ability to remain attune to the healthcare requirements of the population presents several challenges for medical schools, including how they incorporate such shifts into an ever-expanding curriculum [24]. Currently, the UCLMS core curriculum consists of seventeen horizontal modules across the six year programme, excluding the iBSC year (Fig. 1- UCLMS Core Curriculum). The first eight modules in Years 1 and 2 cover basic sciences, with the remaining seven subdivided between the thirty-three hospital-based specialties in Years 4–6. The final two modules in Year 6 are for electives and GP assistantship. In addition, there are sixteen vertical modules that feature as part of Clinical and Professional Practice (CPP) throughout the programme.

The ability to clearly outline the ‘formal’ curriculum or syllabus (Fig. 1- UCLMS Core Curriculum) and demonstrating how it links to the ‘taught’ or ‘assessed’ curriculum is important for both students and educators alike. Increasingly, using Curriculum Maps (CM) to demonstrate these connections has been recognised [25].

### What are curriculum maps?

CMs are centralised resources that provide a visual representation of the different components of the curriculum [25]. By explicitly outlining and linking required intended learning outcomes (ILOs) with content, learning resources and assessment, clarity and transparency is improved [25, 26]. Moreover, enabling students to note connections and relevance to what is being taught, promotes self-directed learning [15, 27, 28]. From a programme governance perspective, CMs can be used to demonstrate quality assurance [5].

### Why develop a curriculum map at UCLMS?

One of the key issues identified from the National Student Survey (NSS) (2018) and student evaluated questionnaires at UCLMS was that many students felt that there was no clear unified syllabus on what they were expected to know and how this could be assessed.

Before producing the CM and at the point at which this study took place, students relied on a mixture of year study guides, the Virtual Learning Environment (VLE) ‘Moodle’ and other online and textbook resources, to access the undergraduate curriculum. However, these resources were disjointed, and ILOs differed depending on which clinical site the students were in placements on. There was no single source of the curriculum. Some students reported feeling disadvantaged in accessing key materials if they were not members of student societies or sports club where it was reported that sharing of assessment material occurred [29]. In order to improve clarity around the syllabus, outline content for assessment, and improve the student experience, the faculty took a strategic decision to create and embed a single source of curricular knowledge: the MBBS Curriculum Map.

Developing a CM, requires careful consultation, planning, building and curation [30, 31]. Specifically, determining the level of detail and complexity is vital as this has implications for costs, staffing and ongoing running. Harden [25] describes the ten ‘windows’ from which a CM can be developed (see Table 1), with the incorporation of additional windows increasing the amount of work required to build and maintain the CM. At UCLMS, a small planning and implementation Curriculum Mapping Team (CMT) was established, consisting of a part-time academic lead, clinical teaching fellow, project manager, and learning technologist (together forming 1.4 full time equivalent).

**Table 1:** The Ten Windows of CM- Developed from Harden (2001)- Curriculum Planning and Building

Window	Description
The expected learning outcomes	Outlines the formal curriculum and will link to windows ‘Curriculum Content’ window and ‘Learning outcomes and ‘Student Assessment’
Curriculum content or areas of expertise covered	Links to ‘Expected Learning Outcomes’ and relates to competency based assessment and knowledge
Student assessment	Learning outcomes- OSCES/ SBAs
Learning opportunities	Links to ‘Learning Resources’ Window and includes independent learning, small group work and large group teaching sessions.
Learning location	Lecture theatres Small group tutorials Laboratory based work
Learning resources	Links to ‘Learning Opportunities’ Window and includes books, articles from journals and simulation
Timetable	Scheduling of learning opportunities in curriculum and can link to ‘Students’ window
Staff	Administrative, professional and technical team involved in maintaining CM
Curriculum management	Relates to team that manages educational activities in curriculum
Students	Includes students portfolio to create personalised learning plan, timetable of activities

One of the first steps taken by the CMT was to clearly identify who the CM was primarily being designed for and their needs [25, 32]. A variety of users were identified here, from students to those involved in medical and clinical education and curriculum planning [33]. However, given the feedback from the NSS, the need to position students as the primary stakeholders of the CM was considered vital at UCLMS. This was to ensure that the CM remained student-centred and aligned to their expectations. It was recognised though that the successful implementation of the CM would require exploring the perspectives of all users, including those involved in medical education and this was an area identified for subsequent development. This study however, details the initial phase of research undertaken by the CMT to scope and understand the requirements from the perspectives of its primary users, the students.

## Methods

This study was conducted at UCLMS with ethical approval granted by the Ethical Committee at the Institute of Education in UCL. All aspects of this research study was carried out in accordance with the guidance and recommendations of the Ethical Committee, with informed consent obtained from the participants prior to data collection.

### Design

To enable the CMT to explore and understand the role and requirements of the CM from a student perspective, an exploratory, sequential mixed-methods research design was used.

The initial phase of the study consisted of gathering quantitative data through a primary survey. This was distributed electronically to all UCLMS students (*n* = 1924) and provided a broad insight from the students’ perspective on the requirements of a future CM. This was followed by seven student focus groups to explore the potential functions and appearance of a CM in more depth.

Two researchers independently used Reflective Thematic Analysis (RTA) to analyse the data collected from the focus groups [34]. Thematic analysis describes the process by which themes are developed using a systematic framework for coding qualitative data [34]. Derived from thematic analysis, RTA follows a less constrained methodology, recognising the researcher’s own biases and position in interpreting and co-constructing these themes through reflective engagement with the data [35].

### Participants

UCLMS has a six year undergraduate programme. Current UCLMS students from Years 1–6 (*n* = 1924), including those in their integrated Bachelor of Science (iBSc) Year 3 were automatically enrolled through the UCL VLE ‘Moodle’ on to the course ‘MBBS Curriculum Map’. Through Moodle, students were sent the link to the online survey, which was active for two weeks.

For the second phase, students were recruited to participate in the focus groups through a message posted on Moodle enabling them to register their interest. Interested students were then sent a Participant Information Sheet outlining the details of the focus groups, and a Consent Form which was completed prior to the focus groups being conducted. All students were aware that participation was voluntary and that they could withdraw at any stage. They were assured that all data collected would be anonymised. This was also reiterated at the start of the focus groups.

To maximise participation across all years, these groups were conducted over lunchtime at the main UCL campus and partner hospital sites, with refreshments provided. As one challenge of focus groups is balancing group dynamics and enabling all participants to contribute so that a breadth of ideas can be captured [36], one focus group was held for each year, so that more junior students would not risk being inhibited by being with senior peers. Recruitment continued until there were between six to ten participants in each group. However, due to attrition on the day, some of the focus groups were smaller (see Table 2). For Year six, an additional focus group was held after an unexpected surge in interest, resulting in seven groups in total.

**Table 2:** Breakdown of Focus Groups

Year	Date	Number of participants	Facilitated by
1	15th February 2019	7	Year 4 UCLMS student
2	21st February 2019	7	Year 4 UCLMS student
3	20th February 2019	5	Year 4 UCLMS student
4	13th February 2019	8	Year 4 UCLMS student
5	13th February 2019	5	Year 6 UCLMS student
6	18th January 2019	8	Clinical Teaching Fellow
6	18th January 2019	4	Clinical Teaching Fellow

### Data collection

#### Online survey

The primary survey was created using online software (Bristol Online Survey, BOS) and consisted of 15 questions divided into three main sections; depiction of content, functionality and students’ likely engagement with a CM (see Table 3). Years 4, 5 & 6 were asked specifically on the inclusion of Core Conditions and Core Presentations on the CM, as these feature on clinical placements only. The final question consisted of an optional free-text response where participants were invited to contribute their own ideas and comments on the CM. The survey was co-created by the Academic Lead and Clinical Teaching Fellow and was piloted on two senior students, as part of the validation process before being distributed across all years. All data received was exported into Excel and anonymised for analysis.

**Table 3:** Online survey

CM	Question	Specific features for inclusion
**Depiction of content**	How important would the following content be in a CM (1=not at all important, 4 = very important)	Core Conditions
		Core Presentations
		Intended Learning Outcomes from their study guides
		SMART Intended Learning Outcomes
		Sign off requirements
**Functionality**	How important would the following features be in a CM (1=not at all important, 4 = very important)	Monitoring progress through the module and year
		Making revision notes
		Uploading files and links from resources
		Linking content to horizontal modules
		Linking content Clinical and Professional Practice (CPP) modules
		Linking content to other years
		Linking content to GMC requirements as outlined by Outcomes for Graduates
**Students’ likely engagement with a CM**	How likely are you to use a CM?	(1=Not at all likely, 4= Very likely)
	What device would you most commonly use to access the CM?	Tablet Laptop Smartphone

#### Focus groups

Each focus group was led by a trained facilitator. It was recognised that students might feel more comfortable if this was facilitated by another medical student. Therefore, two senior UCLMS students were trained by the academic lead for the CMT, who had experience in running focus groups. Training consisted of outlining how to manage dynamics within focus groups and facilitate participant input through the use of probes and prompts. These sessions also included the opportunity to pilot the ‘script’ developed by the Academic Lead on the students, as well as discuss issues pertaining to confidentiality.

While the first two focus groups for Year 6 were led by the clinical teaching fellow from the CMT, the remaining five were led by the senior UCLMS medical students. A member of the CMT attended at the start of each focus group to answer any questions and to check that participants had read the Participant Information Sheet and completed the Consent Form. CMT members not involved in facilitating the focus group then left the room, so as not to risk inhibiting the conversation.

In order to ensure standardisation in the focus groups, facilitators followed a script consisting of five questions with optional, additional prompt/probe questions (Table 4). This script was developed by the CMT and included questions about how students thought the CM could be utilised, as well as the advantages and challenges of its implementation.

**Table 4:** Script for Focus Groups

Question	Prompts & probes
What are your initial thoughts on a curriculum map?	Do you understand what a curriculum map is? What is your vision of what it would look like?
What could you use a curriculum map for?	How do you see it functioning? How do you see it organised? What features would be important to you?
What advantages do you see with a curriculum map?	What would encourage you to use a curriculum map?
What challenges do you see with a curriculum map?	What would stop you from using a curriculum map?
Is there anything that we haven’t touched upon that is important to you?	Is there anything else that you would like to add?

These sessions were audio recorded and transcribed by a professional rapid transcription company, ‘Way with Words’, as stipulated in the ethics form and data protection section. These transcriptions were data cleaned and proof-read by two independent researchers prior to data analysis. Using Reflective Thematic Analysis, all data was analysed inductively with descriptive codes generated from line-by-line coding, before then being grouped together to form themes. To build rigour into the study, each transcript was reviewed by two members of the CMT independently to ensure coherence and alignment in the themes identified.

## Results

### Online survey

**Fig. 2:**
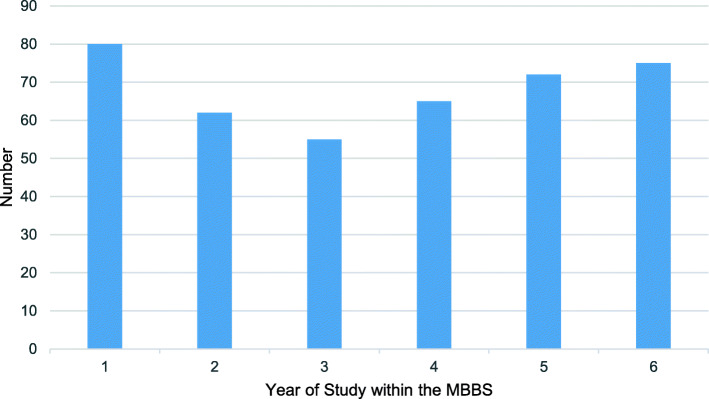
Responses from Online Survey on CM at UCLMS

There were 409 responses from the online survey, representing 21% of the study body (409/1924). Years 2 and 3 had the lowest response rate compared to the other years (Fig. 2- Responses from Online Survey on CM at UCLMS).

Of the students that responded, 92% (377/409) responded that they were ‘likely or ‘very likely’ to use the CM, with a laptop or computer being the most popular choice for accessing the CM followed by smartphones.

**Fig. 3:**
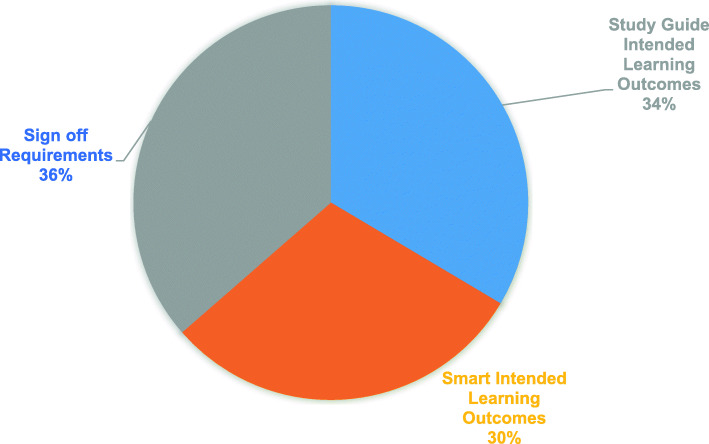
‘Most important’ content feature for inclusion in CM across all years

Across all years, in terms of depicting content, the ability to ‘sign off requirements’ appeared to be the most important feature for inclusion in the CM (Fig. 3-‘Most important’ content feature for inclusion in CM). This was followed closely by the inclusion of ILOs from the study guides but ensuring that these were ‘SMART’ did not appear to be as important. Students in their clinical years, (Years 4, 5 & 6; *n* = 211) were specifically asked on the inclusion of ‘Core Conditions’ and ‘Core Presentations’ from their speciality specific study guides. 91% (192/211) and 84% (178/211) of students respectively responded that the inclusion of ‘Core Conditions’ and ‘Core Presentations’ were ‘very important’ for the CM.

**Fig. 4:**
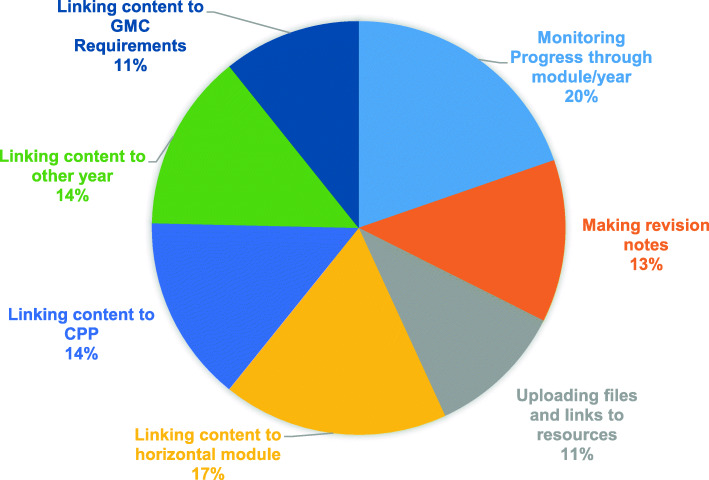
‘Most Important’ functionality feature for inclusion in CM across all years

In terms of functionality, the most important aspect for inclusion in the CM across all years was the ability to monitor progress through the module/year (Fig. 4- ‘Most important functionality feature for inclusion in CM across all years). This was closely followed by content being linked to the seventeen horizontal modules that comprise the core curriculum at UCLMS (Fig. 1- UCLMS Core curriculum). The ability to visualise the connections between the basic sciences modules in Years 1 and 2 to the speciality based modules in the clinical years (Years 4, 5 &6), as well as to the sixteen vertical modules in professional practice were also identified as important by the students. However, linking content to the regulatory requirements as outlined in the ‘Outcomes of Graduates’, or being able to make or upload revision notes or files were considered by students as being less important for inclusion in the CM.

### Themes from RTAs- features considered important by students for inclusion in CM

Five key themes were identified by students across all years as essential for incorporation into the CM. These were gathered from both free-text comments in the survey and from focus group data: both datasets were thematically analysed to synthesise the themes.

The five key themes were that students wanted a CM to be: comprehensive; simple and intuitive; able to link content throughout the course; aligned with assessment; and useful to monitor students’ progress. These are discussed in greater detail, below:

i) ***Comprehensive***

One of the key student preferences was the need for the CM to be comprehensive, in explicitly outlining not only what the students needed to know but with a level of depth of detail. This would determine whether the CM was therefore trustworthy. Whilst it was identified that the CM:

*‘had to be different from the current endless lists of conditions we're supposed to know’*

There was this clear perception of the CM providing a succinct *‘syllabus’:*

*‘The first step should be to just give us a better idea of what our curriculum is and what it is we're supposed to know’*

*‘ … a really detailed curriculum with information on what exactly we are required to know [for assessment]’*

For students on clinical placements, this also incorporated addressing the lack of standardisation due to historical variations in study guides across different sites:

*‘a set of standardised objectives/ learning points that should be achieved by all students across all three hospitals’*

Integral to this requirement of the comprehensive nature of the CM was that it also had to be reliable and contemporary. Interestingly, the limitations and challenges of producing a ‘*be-all-and-end-all list*’ of what the students needed to know, was recognised as unrealistic by a number of Year 6, final year students in the context of the vast and unlimited nature of the discipline of Medicine:

*‘There just needs to be a good solid guide for what is and is not expected of us to be learned. I appreciate that it's impossible to do this’*

Otherwise the need for a clear syllabus,

*‘Something that actually makes it clear what we need to know and in how much detail’*

was universally acknowledged as being a key requirement by the students for the CM.

ii) ***Simple and intuitive***

While it was clear that the students wanted a CM with a definitive set of objectives, equally important was how this information was presented. The need for the CM to be ‘*user-friendly*’was considered essential to its use and success;

*‘Making sure that the tool is as clear and straight-forward as possible will be really important.’*

*‘Students will be more likely to use it if it is intuitive and works well.’*

This notion of ‘*simplicity*’, with ‘*easily accessible content*’, with ‘*direct links to additional resources*’ was universally raised across all years. In addition, the ability ‘*to make notes*’ or ‘*upload files’* onto the CM was also highlighted by several students across all years as a way of making the CM bespoke to individual users.

iii) ***Able to link content throughout the course***

The need for the CM to provide a means to link content across years of the MBBS Programme was highlighted by a number of students in both the focus groups and in the survey free-text comments:

*‘It should be an integrated tool allowing students to see what they are being taught in their year and how it links to learning which was covered [before]’*

This was felt to be lacking in the present system, with some students struggling to relate the relevance of what they were being taught in their basic sciences years (Years 1 and 2) to clinical practice:

*‘There’s an overwhelming amount of information to know, it would be great to see how everything links together’*

Student***s*** also mentioned the importance of effective technical presentation within a CM in visualising these links to augment their learning and maintain motivation:

*‘It would be great to have an interactive visualisation tool to see how curriculum from earlier years is relevant to clinical years … This could potentially improve student experience … motivating pre-clinical students by demonstrating the importance of what they are learning.’*

iv) ***Aligned with assessment***

The desire for the CM to aid in assessment preparation was borne out strongly in the data. A key theme raised by students was the need for the CM to address the perceived lack of alignment between what was being taught and what was being assessed:

*‘I have often found in the past that knowledge covered very briefly and without much emphasis in lectures crops up as a larger proportion of exam questions than one would expect.’*

A number of students felt that the pre-existing system of study guides and VLE resources did not provide adequate guidance for this. Consequently, several students mentioned how they relied on senior peers to aid with this aspect of their learning. It appeared that there was not only a lack of understanding on the depth of detail that they needed to learn but also on how content could be potentially examined. They therefore alluded to a ‘stratification’ system for MBBS content:

*‘It is important to pick out the parts that you need to know for definite and kind of filter through what's less important’*

Interestingly, there was this strong notion of the students not wanting to ‘*waste time learning unless it was relevant*’ and for a CM to clearly highlight what was essential or ‘*core*’ to their learning and assessment. Many echoed this notion of the CM reflecting *‘the potential content of future assessments’* as an aid to enable ‘*students to structure their revision’.*

v) ***Useful to monitor students’ progress***

A further theme to emerge from the data was that the pre-existing study guides did not allow for learning against the course ILOs and core conditions. The ability to use the CM to monitor and chart individual’s progress, was acknowledged as being important by students across all years.

*‘I think it would be a useful tool for students to have their progress through the course (and course material) all in one place, and all displayed in a visually conceivable way’*

Suggestions on how this could be achieved within the CM included, *‘checkbox options’* or a *‘traffic light colour code them … to show myself gaps’* which could be ‘*structured into years and then into horizontal modules and then include key sign off requirements’.*

This idea of the CM operating as a centralised resource where students could track their progress through modules and sign-off requirements for procedures was echoed across the years.

## Discussion

CMs are rare in undergraduate medicine programmes and this, to our knowledge, is the first paper to outline a rigorous approach to approaching the design and build of such a pedagogical device. By exploring the requirements of the CM from the students’ perspective, this paper highlights the importance of building a student-centred CM for ensuring subsequent optimal engagement. Adherence to a CM from both student and faculty perspectives, lays the groundwork for clarity and transparency in assessments, and trustworthiness between students and staff, including clinical teachers in placements.

Essentially, students identified with the theoretical construct of curriculum in the form of a syllabus. Through the data gathered in this mixed-methods study, students told us that the CM’s future success lay in not only providing a comprehensive and detailed list of examinable content, but also structuring it in a way that was accessible and appealing to them. These findings were similarly seen in a curriculum mapping study involving undergraduate pharmacy students in America [37]. Their focus was predominantly on providing a CM that equipped them in preparedness for assessment, and qualification as a fit-for-practise Foundation Year doctor.

However, this study raised a number of issues, including how to manage students’ expectations around the production of an ‘all-encompassing’ CM, in the context of limited time, costs and people-power to design and build it. There was an overall lack of appreciation by students of the complex interplay of factors that could limit its function; in essence we were clear that we were not building a *‘UCLMS Textbook’* and that the CM would need on-going curation to reflect changes within medicine and medical education. From an institutional perspective, setting expectations around the CM as providing a framework for learning (as opposed to an exhaustive textbook) was important. Interestingly, students in the early years of the programme years were more likely to identify a CM as providing didactic information about what to learn and what not to learn, similar to the A-Level specifications that they had become familiar with. While this is perhaps unsurprising, it does raise the importance of addressing the cultural changes that occur in the learning environment transition from school to university. Supporting and empowering students through this transition to develop the skills and values needed for life-long learning is important, especially given how our understanding on human development and illness is constantly changing [38] and that much of medicine is learned through the hidden curriculum.

Another key issue reinforced by this study was students’ perception that learning is principally for passing exams (‘assessment drives learning’), as has been borne out in many studies throughout education and medical education in the past [29]. Assessment forms a critical component for many courses of study but specifically in the MBBS programme, it ensures an appropriate level of competency and safety for students entering the profession [5]. However, this should not undermine teaching delivered on topics that have been traditionally more challenging to directly examine, such as the ‘soft skills’ that are vital for doctors and other healthcare professionals. These include communication skills, professionalism and empathy (which fall under CPP at UCLMS): factors that were woven in to the rubric of our CM design. If one considers that the fundamental role of a medical school is to prepare students for working life, then broadening perspectives beyond passing exams is critical to this [39].

A number of limitations need to be acknowledged in this study. Firstly, the data collected were from students who were willing to participate, and as such, their perspectives may not be fully representative of the entire student cohort. The data collected from the focus groups to enrich and delve deeper into understanding what had been identified initially from the survey (that functioned as a ‘barometer’ of opinion), meant that the self-selecting nature of participants potentially limits the transferability of data from such studies. Secondly, whilst this study focused on the student perspective and was useful in surfacing their opinions, it did not explore the views of other stakeholders, such as clinical teachers and administrative staff. While this has been described in other disciplines, where the CM has primarily been developed from the teacher’s perspectives, one of the risks with this approach is of misaligning the teacher and student’s expectations on the goals of the programme [40]. However, it was acknowledged that this was an area of subsequent work that would be required to ensure the successful implementation of the CM and would include all those identified as secondary users of the CM.

Managing expectations of how the CM would function was therefore crucial, as logistically incorporating all of these features would be challenging. This was especially pertinent within the context of the timeframe available for building and testing the CM before implementation. In particular, there were limitations regarding the ability of students to see and sign off practical procedures within the CM, which was an original aim of the CMT, but did not come to fruition in the time envelope. It was acknowledged that this would require an immense amount of work, beyond the scope of this phase of the project. However, it was acknowledged that over time the CM would evolve and that part of the CMT’s role would be in adapting and incorporating amendments in the CM.

Finally, while medical students were identified by the CMT as the primary users, considering the viewpoints of UCLMS professional support staff, teachers, and stakeholders was equally important. The plan going forward for the CMT was to liaise with the year and specialty leads in developing the content and links to learning resources in the CM. The aim being that both students and educators alike would have access to a more robust framework, in the form of the CM, for accessing information.

## Conclusion

The decision to create and develop a CM at UCLMS in response to a combination of student feedback and metrics was a significant undertaking. For the CMT, the first step in this process involved understanding the requirements of the CM from the perspective of the primary stakeholders: medical students. In doing so, the CMT were able to identify the key needs for this centralised resource that would not only be simple to access and use but also could be personalised to the user. This study highlighted certain limitations and the need to set realistic expectations around the function of an electronic syllabus, or CM. Nevertheless, by establishing the core requirements at an early stage from its primary users, it provided direction and scope on the development of the CM. This study has provided a springboard to develop a student-centred, workable MBBS Curriculum Map.

## Data Availability

The datasets generated and/or analysed during the current study are not publicly available to protect participant confidentiality but are available from the corresponding author on request.

## Abbreviations

BOS: Bristol Online Software
CM: Curriculum Map
CMT: Curriculum Mapping Team
CPP: Clinical and Professional Practice
GMC: General Medical Council
ILOs: Intended Learning Outcomes
MBBS: Bachelor of Medicine, Bachelor of Surgery
NSS: National Student Survey
UCL: University College London
UCLMS: University College London Medical School
VLE: Virtual Learning Environment
